# Metal/Metalloid-Based Nanomaterials for Plant Abiotic Stress Tolerance: An Overview of the Mechanisms

**DOI:** 10.3390/plants11030316

**Published:** 2022-01-25

**Authors:** Mohammad Sarraf, Kanchan Vishwakarma, Vinod Kumar, Namira Arif, Susmita Das, Riya Johnson, Edappayil Janeeshma, Jos T. Puthur, Sasan Aliniaeifard, Devendra Kumar Chauhan, Masayuki Fujita, Mirza Hasanuzzaman

**Affiliations:** 1 Department of Horticulture Science, Shiraz Branch, Islamic Azad University, Shiraz 71987-74731, Iran; sarraf.science@gmail.com; 2 Amity Institute of Microbial Technology, Amity University Uttar Pradesh, Noida 201313, India; kvishwakarma@amity.edu; 3 Department of Botany, Government Degree College, Ramban 182144, India; vinodverma507@gmail.com; 4 D. D. Pant Interdisciplinary Research Laboratory, Department of Botany, University of Allahabad, Prayagraj 211002, India; namirarif@gmail.com (N.A.); profdkau@gmail.com (D.K.C.); 5 Plant Physiology and Biochemistry Laboratory, Department of Botany, University of Calcutta, Kolkata 700019, India; sdbot_rs@caluniv.ac.in; 6 Plant Physiology and Biochemistry Division, Department of Botany, University of Calicut, C.U. Campus P.O., Kozhikode 673635, India; riyajohnson@uoc.ac.in (R.J.); edappayiljaneeshma@gmail.com (E.J.); jtputhur@yahoo.com (J.T.P.); 7 Photosynthesis Laboratory, Department of Horticulture, Aburaihan Campus, University of Tehran, Tehran 33916-53755, Iran; aliniaeifard@ut.ac.ir; 8 Laboratory of Plant Stress Responses, Faculty of Agriculture, Kagawa University, Miki-cho, Kita-gun, Kagawa 761-0795, Japan; 9 Department of Agronomy, Faculty of Agriculture, Sher-e-Bangla Agricultural University, Dhaka 1207, Bangladesh

**Keywords:** abiotic stress, plant stress tolerance, metalloids, metalloid nanoparticle, antioxidant enzymes, antioxidant defense, ascorbate peroxidase, glutathione reductase, reactive oxygen species

## Abstract

In agriculture, abiotic stress is one of the critical issues impacting the crop productivity and yield. Such stress factors lead to the generation of reactive oxygen species, membrane damage, and other plant metabolic activities. To neutralize the harmful effects of abiotic stress, several strategies have been employed that include the utilization of nanomaterials. Nanomaterials are now gaining attention worldwide to protect plant growth against abiotic stresses such as drought, salinity, heavy metals, extreme temperatures, flooding, etc. However, their behavior is significantly impacted by the dose in which they are being used in agriculture. Furthermore, the action of nanomaterials in plants under various stresses still require understanding. Hence, with this background, the present review envisages to highlight beneficial role of nanomaterials in plants, their mode of action, and their mechanism in overcoming various abiotic stresses. It also emphasizes upon antioxidant activities of different nanomaterials and their dose-dependent variability in plants’ growth under stress. Nevertheless, limitations of using nanomaterials in agriculture are also presented in this review.

## 1. Introduction

The upcoming challenges of rise in global population, decreasing arable lands, and escalating threats posed by climate change exert pressure on the need for developing new techniques and methods to increase yield potential during stressful conditions. Stressful conditions for plants arise from numerous biotic and abiotic factors, which impart stresses such as drought, salinity, temperature, and heavy metal leading to substantial modifications in plants. Thus, improving stress tolerance in crops is a major target of research to fulfill the food demand of growing populations. Over the last several decades, tremendous efforts are being taken to improve the agricultural yields through extensive application of chemicals that have long-lasting and profound effects on the environment and human health. Therefore, to feed the world population without damaging the environment, the application of novel technology is necessary.

Nanotechnology is a novel approach towards the improvement in the agricultural sector as it puts forth new ways to impart tolerance against various stresses and enhances the productivity [1]. Nanoparticles (NPs) are molecules with dimensions of 100 nm, diverse physicochemical properties, higher reactivity, and biochemical activity which depends on their high surface energy and the high surface-to-volume ratio [2]. Plants have the ability to synthesize NPs which are natural agents used for improving the morphology of the plants without imparting any negative effects [3]. In the current situation, NPs have the potentiality to boost plant morphogenesis, used as herbicides, nanopesticides, and nanofertilizers, etc., that can proficiently release their content in required amounts to target cellular organelles in plants. Still, certain potentials of NPs are not revealed due to a lack of mechanisms that are not cleared or nor yet studied.

Different types of NPs are developed such as those containing inorganic nonmetallic NPs, carbon-based NPs, metallic NPs, and organic polymeric materials based on the application and usage [4]. Effective nutrient supply requires specific nanofertilizers or nanoencapsulated nutrients that could act as an efficient tool towards sustainable mode of agricultural practices. These nanofertilizers would be an alternative to chemical fertilizers that, in turn, improve efficiency of resource utilization, reduce soil toxicity, and thus, usage of nanofertilizers will assist to diminish such problems [5]. Plants are sessile so they have to face extreme environmental stress conditions, such as salinity, drought, high and low temperatures, heavy metals, flooding, high and low light intensities, ultraviolet (UV), and others. The extreme environmental conditions induces bursts of reactive oxygen species (ROS) which causes macromolecules and membrane degradation, prompts cell toxicity, and diminishes the plant growth. Antioxidant machinery through enzymatic and non-enzymatic systems scavenges ROS to alleviate oxidative stress. Against various abiotic stress, NPs take part in the growth and development of plants followed by providing protection to plants [6]. NPs have the capability to modify those genes (and their expressions) that are involved in cell biosynthesis and organization, electron transport, and energy transport during stress responses [2]. From different experiments, it was concluded that NPs play a very important role in improvement of crop plants, but understanding of the appropriate mechanism [1,7,8,9,10] and the way of interaction of NPs with plants at different levels is still required at an early stage. Current review focuses on the concept, types, mode of metal/metalloid nanoparticles together with physiological impact of metalloid NPs on plants, their effect on growth and overcoming abiotic stress, and the underlying mechanisms.

## 2. Concepts and Types of Nanoparticles

The use of NPs has a novel approach, which allows a better understanding of interconnection of science and technology, and opens up new interventions in the field of biotechnology and agriculture [11]. Particles having dimensions between 1–100 nm are considered as NPs; they have high surface vitality and large surface to volume ratio that increases their reactivity [12]. Besides having small dimensions and high reactivity, each NP contains its unique physical and chemical properties. They are composed of three layers: the outer layer known as surface layer, middle layer known as shell layer, and the inner layer is called core layer. The shell layer is found chemically different from core layer [13]. In the present scenario, which depicts indulging of various materials and novel techniques to create a boom in agricultural crops and in improving crop quality, the application of NPs in the agriculture field shows potential results through increasing plant growth and production, as different NPs are applied through various methods, for instance, as herbicides, nanopesticides, nanofertilizers, etc. [14]. The major difference between mode of action of other elements and NPs in plants is that NPs are effectively released in required amounts and reach the targeted cellular organelles [12]. Although, despite having numerous initial studies on potential application of nanomaterials to attain the objective of flourishing agriculture, there is still a need to unfold their unique mode of action in plant system, which helps to boost the agriculture production one level up [15].

NPs have different sources of origin, namely natural, incidental, and engineered [16]. Natural occurrence of NPs is from volcanic eruptions, dust storms, mineral complexes, forest fire, photochemical reactions, etc. Incidental origin of NPs occurs through human interventional activities, such as exhaust from metallurgic activities, coal combustion, and industries [16]. Whereas, engineered NPs are generally classified into carbon-based NPs, metal-based NPs, metal magnetic NPs, dendrimers, and composite NPs. Metal and metal oxide-based NPs from the past several decades are comprehensively studied in agriculture field for the improvement of crop productivity, and increasing the plant resilience and tolerance under abiotic stress conditions [17]. Metal-based NPs include nanomaterials of gold (Au), silver (Ag), copper (Cu), aluminum (Al), and iron (Fe). Additionally, their oxides, such as titanium dioxide (TiO_2_), cerium oxide (CeO_2_), iron oxide (FeO), aluminum oxide (Al_2_O_3_), and zinc oxide (ZnO) are also gaining so much attention of scientists worldwide to tackle adverse environmental conditions [18,19,20]. The different types of nanoparticles are given in Table 1.

## 3. Synthesis of Metal and Metalloid Nanoparticles

The synthesis of metal and metalloid NPs is a promising part of nanotechnology, which offers solutions for wide areas including agriculture [33]. Engineered NPs have distinctive electrical, mechanical, physiochemical, optical, and imaging properties that can be controlled during synthesis process [34]. The difference between metal/metalloid NPs and their bulk material occurs on the basis of size, shape, and surface characteristics, such as presence of coatings, copious reactive sites, and mobility regulated by their aggregation state [35] that further depends on their pH, temperature, ionic strength, and concentration [36]. So far, a number of methods have been developed for controlled synthesis of NPs. Generally, there are two main approaches such as: (i) bottom-up approach and (ii) top-down approach [37]. These are further classified under many subclasses developed on the basis of operation, reaction condition, and adopted protocols.

Top-down pathway includes synthesis by gradual size reduction, which is achieved via various physical and chemical methods [38]. In general, it operates when particles are larger than nano-sized particles [34]. Whereas, in bottom-up means of synthesis, NPs are produced from atoms and molecules that include reduction/oxidation as core reaction [39]. This pathway is followed when metal particles are already smaller than nano-sized molecules. During synthesis, NPs aggregate through the action of reducing agents which also act as anti-agglomerating agents [34]. Plant extracts and chemicals act as reducing agents, as they contain alkaloids, terpenoids, flavonoids, phenols, carbohydrates, anthraquinones, and proteins, etc., which reduce the size of metal ions into NPs and stabilize the resultant NPs [40].

**Figure 1:**
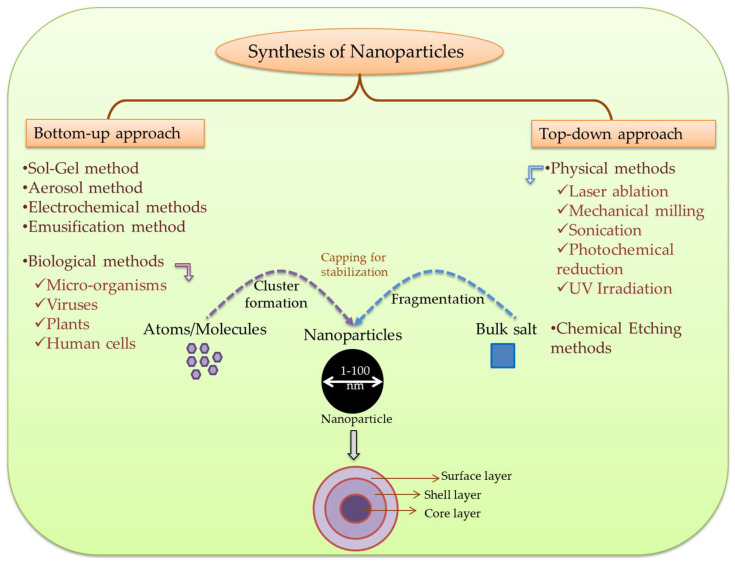
An overview of nanoparticles’ synthesis.

Moreover, bottom-up approach follows the involvement of biogenic substances. Biological agents required for the synthesis are bacteria, yeast, algae, cyanobacteria, fungi, flagella, viruses, plants, and even human cells [41]. For the reducing agent, microorganism and plant extracts are used [42]. Biological synthesis is more feasible, cost-effective, ecologically-friendly, and less toxic to the environment [41], due to their distinct optical, chemical, photoelectrochemical, and electronic properties [43]. A wide range of physical, chemical, and biological methods including environment-friendly green synthesis of NPs are developed and applied in various disciplines. The size of NPs can be manipulated by controlling various parameters such as pH, temperature, concentration, and exposure time to substrate [34]. For instance, a method was developed to manipulate the shape and size of AuNPs extracellularly produced by microorganisms through shifting the key growth parameters [43]. Some study shows that AuNPs’ synthesis occurs by using the plants rich in tannic acid, whereas to synthesize AgNPs, chemicals like trisodium citrate can be used as important catalysts [44,45]. The overview of nanoparticles’ synthesis is illustrated in Figure 1.

## 4. Mode of Action of Nanoparticles in Plants

**Figure 2:**
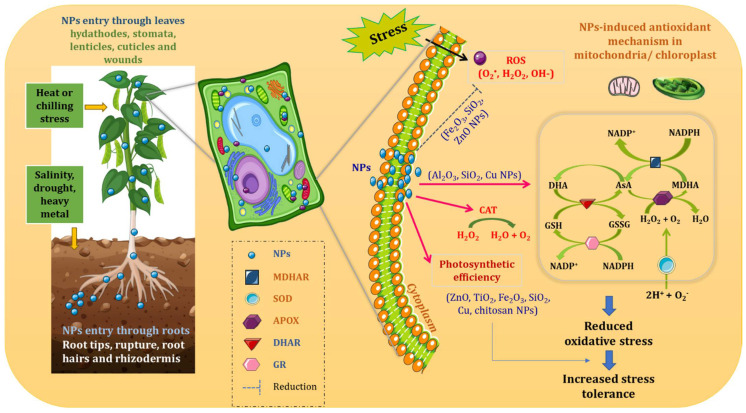
Antioxidative mechanism of action of nanoparticles in plants under abiotic stress (NPs: nanoparticles; MDHAR: monodehydroascorbate reductase; SOD: superoxide dismutase; APOX: ascorbate peroxidase; DHAR: dehydroascorbate reductase; GR: glutathione reductase; ROS: reactive oxygen species).

Several hypotheses have been made from the studies that were conducted to know the exact NPs’ mode of action (Figure 2). Certain studies showed that NPs which mediated growth of plants depends upon the concentration of NPs utilized; this can be toxic to plant growth at higher concentrations [46,47,48] or it can be beneficial when given in relevant concentrations [49,50]. Entry of NPs into the cells happens either by penetration or by transportation via particular channels located in the cellular membrane. NPs might function as stress signaling molecules which, in turn, cause induction in the expression of various genes involved in stressed condition. This includes the induction of expression of regulatory factors thus resulting in activation of defense system, and finally, exhibiting stress tolerance. Besides an acceptable level, NPs can maintain ROS at considerable level to induce ROS signaling network hence activating defense system of plant under stress conditions. Ruotolo et al. [51] performed meta-analysis of proteomics and transcriptomics studies where the response of different plant species to metal-based NPs was compared. It was found that common NPs which induced responses to stress include root architecture modification, antioxidant mechanism activation, and involvement of specific signaling pathway of phytohormones, although the effects were influenced by NPs’ nature and their duration of exposure [51,52]. For example, after exposure to NPs, the root architecture modification could be due to the downregulation of genes involved in trichoblast differentiation. This is the area from where the emergence of root hairs occurs hence trichoblasts come under specialized epidermal cells. Further, genes responsive to indole acetic acid (IAA) and ethylene (ET) were shown as the positive regulators of development of root hairs [51]. NPs’ treatment frequently alters biological pathways involved in defense mechanisms [51]. NPs’ treatment also upregulates genes that encode for proteins which play a primary role in ROS balance like NADPH oxidase, GST, superoxide dismutase (SOD), and peroxidases (POX) [51].

The genes responsible for activation of antioxidant enzymes are upregulated by NPs [53]. Laware and Raskar [53] carried out an experiment to determine the effects of TiO_2_ NPs on onion seedlings, and from the results, they suggested that the activity of SOD enzyme was elevated by TiO_2_ NPs where the enzyme’s activity was further enhanced when the concentration of NPs was increased. However, only at low concentration of TiO_2_ NPs, there was an improvement in seedling growth and seed germination in onion which was suppressed at high concentration of TiO_2_ NPs [53]. One study showed an enhancement of seed germination and growth in *Glycine max* seeds when exposed to TiO_2_ and SiO_2_ NPs [54].

The studies also reported that NPs can be recognized by calcium-binding protein (CaBP) complex or as signaling molecules in the cytoplasm. Once NPs enter plant cells, NP-specific proteins are recognized which then triggers the downstream expression of stress-related genes [9,55]. As a result, a cascade of signaling pathways is induced intracellularly, and associated genes are upregulated whose expressions lead to plant’s increased tolerance responses to adverse environmental conditions. When *Arabidopsis thaliana* was exposed to salinity and drought conditions or treated with ABA, responsive to desiccation (RD20) gene expression was induced which harbors a specific conservative region for binding of calcium ion (EF-hand) [56]. In a study, increase in the expression of RD20A, particularly in Co and Fe NPs-supplemented plants, supported the hypothesis that NPs take part in induction of Ca^2+^- binding protein expression [55]. Besides that, NPs are also thought to impart a vital role in scavenging ROS by inducing the activities of antioxidant enzymes. Recently, very strong evidence was provided by Sun et al. [57] which shows that the expression of Cu/Zn SOD, Fe/Mn SOD, catalase (CAT), and ascorbate peroxidase (APX) was notably enhanced in plants that were treated with ZnO NPs under drought.

Various transcriptomics and proteomics studies have been carried out to assess plant and nanomaterial association [10]. Results from transcriptomics studies showed the effects of (≤50 nm size) Cu-based NPs which modulate the genes responsive to oxidative stress, brassinosteroid biosynthesis, and root formation [58]. Metabolomics studies on 40 nm sized Cu NPs in cucumber (*Cucumis sativus*) showed increase in secondary metabolite (such as acetyl glucosamine, phenyl lactate, 4-aminobutyrate) accumulation involved in cell signaling and defense responses, and decrease in metabolites of flavonoid and fatty acid synthesis, as well as riboflavin and amino acid metabolism [59]. Moreover, TiO_2_ NPs- treated tobacco plants had a significant elevation in transcript levels of miR399 and miR395 in transcriptome analysis, both of which are involved in regulation of adaptive responses of plant to nutrient stress, thus suggesting the fact that these miRNAs in tobacco plants have a significant role in responding to TiO_2_ NPs [60]. When the seedlings of *A. thaliana* were exposed to carbon nanodots of 3 nm, root elongation happened in a dose-dependent manner; transcriptomics analysis revealed that the genes involved in cellular response to phosphate starvation, UDP-glycosyltransferase activity, and stimulus response were upregulated whereas those which took part in chloroplast structure and function were downregulated [61]. Results from metabolomics study suggested the occurrence of defense response activation due to the augmentation of cell wall’s carbohydrate components.

### Metal/Metalloid-Based Nanoparticles for Enhancing Plant Antioxidant Defense

Antioxidant defense system of plants comprise of various enzymes like CAT, APX, dehydroascorbate reductase (DHAR), guaiacol peroxidase (GPX), glutathione reductase (GR), and SOD and low molecular weight antioxidant compounds such as glutathione and ascorbate (Figure 2) [62,63]. It has been confirmed that enzyme-like activities are possessed by various NPs where nCeO_2_, nFe_3_O_4_, nCo_3_O_4_ NPs imitate CAT; nCeO_2_, nFe_3_O_4_, nCo_3_O_4_, nMnO_2_, nCuO, and nAu mimic peroxidase; nCeO_2_, nPt, and fullerene mimic SOD activity [62]. With all this information in hand, still, efficient techniques are required to detect enzymes mimicking activities of NPs when supplemented to the whole plant.

Maghemite γ-Fe_2_O_3_ nanomaterials (NMs) and magnetite Fe_3_O_4_ NMs are the most common forms among ferromagnetic FeO NMs [64,65,66]. It was first unveiled by Gao et al. [67] that Fe_3_O_4_ NPs have POD-like activity and the results showed that with decreasing Fe_3_O_4_ NPs particle size, the catalytic activity would be significantly increased [67,68]. In Fe_3_O_4_ NPs, the Fe is present in either ferrous (Fe^2+^) or in ferric (Fe^3+^) form where the POD-like activity is higher when NPs are in ferrous Fe^2+^ form [67]. Chen et al. [64] proved ferromagnetic FeO NPs can also act like CAT enzyme thus owning dual enzyme-like activity property. At an acidic pH of 4.8, hydrogen peroxide is catalyzed by ferromagnetic FeO NPs forming ^•^OH thus exhibiting POD-like activity, whereas at neutral conditions ferromagnetic FeONPs exhibit CAT-like activity, decomposing hydrogen peroxide to H_2_O and O_2_. Side-by-side comparison of catalytic performance was done on two types of FeO ferromagnetic NPs on the basis of surface charge and similarity in sizes. From the results, it was known that POD-like activity was possessed by Fe_3_O_4_ NPs than γ-Fe_2_O_3_ NPs [64]. From all these, it can be concluded that ferromagnetic FeO NMs can perform multifunctional activities by combining enzyme-like and magnetic properties. In a study, doping γ-Fe_2_O_3_ NPs with yttrium has decreased the amount of H_2_O_2_ by 45% and peroxidation of membrane lipid by 28% in the leaves of *B. napus*, leading to alleviation of drought stress impacts on plant [69]. When maize grown in calcareous soil was foliar-sprayed with Fe_3_O_4_ NPs, scavenging of H_2_O_2_ was enhanced, and the rate of peroxidation of membrane lipid was brought down in comparison to the control [70]. Similarly, Fe_3_O_4_ NPs have been used to protect cadmium toxicity in tomato plants by reducing oxidative stress level [71]. Using all these results, it can be confirmed that γ-Fe_2_O_3_ and Fe_3_O_4_ NPs protect plants from environmental stresses. In addition to that, Li et al. [72] carried out an experiment in seedlings of *Citrus maxima* to compare γ-Fe_2_O_3_ and Fe_3_O_4_ NPs. It was found that Fe_3_O_4_ NPs have more antioxidant capacity than the γ-Fe_2_O_3_ NPs.

CeO_2_ NMs are considered as the initial NMs, which have SOD-like activities exceeding the catalytic activity of native SOD [73]. The preliminary mechanism to possess enzyme-like activity is to have the ability to switch between two valence states (Ce^3+^ and Ce^4+^) with a significant level of oxygen vacancy on its surface [74]. CeO_2_ NMs retains longer when the cycling is between two oxidation states (Ce^3+^ and Ce^4+^) and remains uninterrupted with Ce^3+^ being continuously regenerated. Various studies have been carried out in the past to determine the multifunctional enzyme activity (SOD and CAT) of CeO_2_ nanozymes [73,75,76]. As a thumb rule, CeO_2_ NMs function as SOD-like when the ratio of Ce^3+^/Ce^4+^ is high and CAT-like when the ratio is low [77]. Under alkaline or neutral conditions, CeO_2_ NMs exhibit CAT-/SOD-like property whereas under acidic conditions OXD-/SOD-like property is exhibited by CeO_2_ NMs [76]. It is henceforth clear that O_2_^•−^ and H_2_O_2_ can be scavenged by CeO_2_ NMs due to their ability to mimic ROS scavenging enzymes. Recently CeO_2_ NMs have attracted attention to scavenge ROS in plants under environmental stresses. The coating of anionic poly (acrylic acid) on CeO_2_ NPs (10nm) with low (35%) ratio of Ce^3+^/Ce^4+^ has been reported to scavenge ROS by 52% in the *A. thaliana* leaves subjected to abiotic stress [78]. Sorghum leaves under drought stress have been compared by spraying water (control) and CeO_2_ NPs to leaves, and it was observed that leaves sprayed with CeO_2_ NPs had decreased O_2_^•−^ content by 41% and H_2_O_2_ content by 36% as compared to control [79]. In cotton roots, efficient reduction in accumulation of ROS by 46% has been observed when seeds were primed with poly (acrylic acid)-coated CeO_2_ NPs under salinity stress [80]. The results of transcriptomic analysis showed that tolerance to saline conditions had improved when seed priming with CeO_2_ NMs had been carried out which induced changes in expressions of gene family coding for antioxidant enzymes [80]. Thus, it is clear from previous studies that CeO_2_ NMs have dual roles of scavenging ROS and are an inducer of antioxidant enzymes.

Cobalt oxide (Co_3_O_4_) NPs have dual intrinsic POD-like and CAT-like enzyme activities [81]. Transfer of electrons between H_2_O_2_ and the substrates potentially offer Co_3_O_4_ NPs the ability to function like POD. Although Co_3_O_4_ NPs have dual intrinsic enzyme-like activities, its ability to function as CAT-like is weaker than that of its ability to function like POD. However, the CAT-like activity can be modified by changing the pH to neutral or to basic from acidic conditions [82]. Jahani et al. [83] did a field work of spraying Co_3_O_4_ NPs at different concentrations, where the foliar spray of these NPs at a concentration <100 mg L^−1^ induced growth of plant and did not cause production of ROS; however, at >250 mg L^−1^ concentration of Co_3_O_4_ NPs, ROS generation was induced and negatively affected growth and photosynthetic activity. It is still a mystery that the plant growth inducing effect of Co_3_O_4_ NMs is because of its ability to act enzyme-like or due to some other unknown function. Future research must be carried out to understand the association between Co_3_O_4_ NMs and plants under environmental stress.

Manganese NMs such as Mn_3_O_4_, MnO, and MnO_2_ have the ability to eliminate high amounts of ROS and also possess enzyme-like activities [84,85,86]. From the study of Ragg et al. [84], it is known that SOD-like activities are exhibited by MnO NPs where the enzyme-like activity is surprisingly greater as compared to native Mn-SOD. However, apart from SOD, multiple other enzyme activities have been mimicked by MnO_2_ such as OXD, POD, and CAT [85]. A very satisfying ROS scavenging efficacy was exhibited by Mn_3_O_4_ NPs where ^•^OH was removed [86]. The fast redox exchange between two oxidative states of Mn (Mn^2+^ and Mn^3+^) is crucial for the intrinsic multifunctional enzyme-like activity of Mn_3_O_4_ NMs [87]. H_2_O_2_ and O_2_^•−^ couple show a high degree of affinity for H_2_O_2_ and O_2_^•−^ than any other transition metal couples. It was also found that Mn_3_O_4_ NPs’ ability to eliminate ROS was way superior to that of CeO_2_ NPs [86]. Hence manganese oxide-based NMs can be used as a promising therapeutic tool for treating ROS-mediated diseases [86,87,88]. Taking into account the abovementioned observations, more relevant studies regarding the catalytic and antioxidant activities of Mn_3_O_4_ NMs are needed in the coming future.

There are some other NPs that can be beneficial at low concentrations but toxic when supplied at higher concentrations. Zinc oxide (ZnO) NPs have been used in plants to overcome Zn deficiency and abiotic stresses. When ZnO NPs with the size of 90 ± 10 nm applied at varying concentration between 400–3200 mg Zn kg^−1^, levels of superoxide (O_2_^−^) radical were found to be elevated and a significant raise in SOD activity at a maximum dose was documented in maize [89]. On treating *Gossypium hirsutum* with ZnO NPs, enhanced POX and SOD activities with a subsequent drop in lipid peroxidation was reported [90]. ZnO NPs come in various shapes and sizes like spherical (38 nm), floral (59 nm), rod-like (>500 nm), and also Zn^2+^ ions; out of all these, the most protective form was found to be spherical ZnO NPs of size 38 nm which elicited the greatest oxidative stress responses (SOD, POX, MDA, CAT, H_2_O_2_ synthesis) in soybean [91].

The pretreatment by TiO_2_, ZnO NPs resulted in obvious increase in GPX and SOD activity which also improved the tolerance against heat stress, further lowering the levels of H_2_O_2_ and causing membrane stabilization (1.5 times) [92]. Gene expression analyses on *A. thaliana* exposed to ZnO NPs showed 660 up- and 826 downregulated genes [93]; further analyses on roots exposed to TiO_2_ NPs and fullerene soot (FS) NPs revealed 80 up- and 74 downregulated genes and 232 up- and 189 downregulated genes, respectively (expression difference > 2-fold). 

Enhanced activities of APX, GPX, CAT, and GR were noticed when seedlings of *Brassica juncea* were treated with gold nanoparticles (GNPs) which also resulted in proline and H_2_O_2_ accumulation in an amount greater than usual in plants treated with GNPs which kept on increasing with increase in concentration of GNPs [94].

Extensive research is still being carried out to understand the interactions between plants and metallic oxide nanomaterials (NMs) [95,96]. Few metal-oxide NMs such as CeO_2_NMs, MnO_2_ NMs, cobalt oxide (Co_3_O_4_) NMs, and ferromagnetic FeO are available in mixed valence state and hence have the ability to function as nanozymes for scavenging free radicals [65,96,97].

## 5. Application of Metal and Metalloid Nanoparticles for Improving Abiotic Stress Tolerance

Abiotic stresses are major problems for agriculture productivity and extension. They include drought, salinity, alkalinity, submergence, mineral and metal toxicity/deficiencies, and many others that reduce crop growth and productivity. Plants adapt and mitigate abiotic stresses by alterations in morphological, physiological, biochemical, and molecular levels to combat various stresses. Researchers have revealed that NPs help plants to overcome abiotic stresses by their concentration-dependent impact on plant growth and development [98]. The effect of abiotic stresses and the ways by which NPs combat abiotic stress and impart tolerance is depicted in Table 2. Recapitulation of the possible interaction between NPs and plant metabolisms is essential to explore the novel insights in the field of plants’ stress tolerance.

### 5.1. Drought

Among different stresses, drought is a frequently occurring stress, causing scarcity of water followed by high temperature and loss of water uptake by the plants. It is mainly found in the dry and semiarid regions thereby affects plant growth at early stage, i.e., starting from seed germination to seed setting [116]. Drought stress can be transformed by different NPs’ application such as studies reported that drought stress tolerance in plants imparted by silica NPs. According to Ashkavand et al. [117], application of silica NPs in hawthorns improved seedling growth and physiological parameters under drought stress. Similar results were observed in *Triticum aestivum*, which improved starch and gluten content thereby improving growth and yield under drought condition [107]. This amendment is due to the ability of TiO_2_ to facilitate germination of seeds and growth of seedlings. TiO_2_ also helps to increase biomass, keep relative water content (RWC), and boost antioxidative enzymes in plants under drought stress [6]. Jute seeds treated with CaNP (hydroxyapatite nanoparticle) showed improved tolerance against drought stress via biosynthesis of proline and thus controlling the level of proline [118]. Although drought stress severely hampers the corn seedlings and decelerates its growth, whereas treatment with yttrium-doped Fe_2_O_3_ NPs improved photosynthetic machinery with increased chlorophyll, carotenoid content, and also ameliorated the negative impacts of drought on *B. napus* [69].

According to Sedghi et al. [119], ZnO in *G. max* improved seed germination percentage and dry weight, by utilizing seed reserves at faster rate due to the increased activity of gibberellins. Similarly, Fe_2_O_3_ enhanced tolerance against drought stress by modifying carbohydrate metabolism and stomatal movements. Studies conducted in maize proved that nano ZnO downregulate photosynthetic pigment degradation and thus enhance the rate of photosynthesis and stomatal movements. Starch and sucrose synthesis were also enhanced by manipulating key enzymes such as UDP glucose pyrophosphorylase, phosphoglucoisomerase, and cytoplasmic invertase leading to better performance under drought stress [57]. This makes ZnO a potential nano agent to mitigate the negative effects of drought stress. Van Nguyen et al. [103] reported that in maize, CuO NPs positively regulate pigment system and ROS scavenging mechanism to cope with drought stress. Application of the same NP at low concentration via roots and leaves has been found to improve crop performance by enhancing the performance of chlorophyll and photosynthetic enzymes such as RubisCO and thereby photosynthesis. It also helps in supplement uptake, fortifying stress resilience, and positively impacts on yield.

### 5.2. Salinity

Salt stress is the most noteworthy universal concern that influences crop growth and productivity. Unusual increase in sodium (Na^+^) and chloride (Cl^−^) generates cytotoxicity and imbalance in nutrition further coupled with oxidative stress due to ROS production followed by implementing a strategy of osmoregulation. During osmoregulation, the plant will accumulate the organic compounds such as amino acids, polyols, sugars, glycine betaine, and quaternary ammonium compounds which further results in decreased osmotic potential. Another key solution is ion homeostasis where the concentration of Na^+^ is reduced and K^+^ concentration will be increased in the cell to overcome the ROS affect and to start the activity of enzymatic machinery [120,121].

NPs help in mitigating such stresses by activating specific genes, accumulating osmolytes, and providing free nutrients and amino acids. In *Cucurbita pepo*, treatment with SiO_2_ NPs improved the plant transpiration rate, water use efficiency (WUE), enzyme carbonic anhydrase activity, and defense response against salinity stress [122]. Correspondingly, TiO_2_ (anatase) alters the photoreduction activity and hinders linolenic acid in the electron transport chain (ETC) [123]. A study carried out in *Abelmoschus esculentus* revealed that foliar application of ZnO improves photosynthetic functionality and enzymatic machinery to reduce negative impacts of salinity stress. It positively impacted on plant growth and resulted in enhanced photosynthesis by improving the efficiency of photosystem II. It also helps to maintain RWC thus decreasing membrane damage [124]. Similarly, combined application of ZnO and Si as foliar spray in mango seedlings augmented the carbon assimilation and nutrient uptake further leading to improved growth conditions [125]. Various reports on SiO_2_ application confirmed improved vegetative growth, increased epicuticular wax layer, accumulation of proline, and salt stress genes were up- or downregulated to mitigate salinity impact in different plants such as *Solanum lycopersicum*, strawberry, and *Ocimum basilicum* [126,127,128].

AgNPs is a well-known nanomaterial; it has been reported that AgNPs act as potential nano agents to mitigate salinity stress. AgNPS in *T. aestivum* increased the accumulation of POD, proline, and sugar, further followed by enhanced germination [129]. Similarly, treatment with CeO, CNTs, and graphene NPs improved the assimilation of photosynthetic carbon, increased the proteins and amino acids at reproductive stage, and thus imparted tolerance against salinity stress in cotton and *Catharanthus roseus* [80,130]. ZnO enhanced salt tolerance by lowering the contents of malondialdehyde (MDA) and Na^+^ in lupine plants, and improved germination in cumin seeds. Application of n-ZnO diminished the negative effects of NaCl through enhancing photosynthetic system, proper osmoregulation, and bringing down the levels of MDA and Na^+^ [19].

### 5.3. Extreme Temperature

Temperatures above maximum threshold level are called heat stress and below a minimum threshold level are known as cold stress. These stresses can create an imbalance of cell homeostasis and promote serious hindrance which may even lead to the death of the plants. Extreme temperature directly imparts a combination of heat, and as a consequence, oxidative stress leading to the excessive production of reactive species and further alterations in physiological and biochemical activity such as production of various osmolytes and heat shock proteins (HSPs) that can protect proteins and cell structures, and enhance antioxidant mechanism to restore the redox potential and homeostasis [131]. 

NPs such as selenium were found to be effective in combating high temperature stress. Djanaguiraman et al. [79] reported that application of selenium NPs in sorghum improved their antioxidant machinery to scavenge ROS produced as a result of heat, thus alleviating heat stress. Similar results of SeNPs were observed in *L. esculentum* that imparted tolerance against both high and low temperature stresses [108]. Photosynthetic apparatus of wheat plants was highly affected by heat, however, use of AgNPs imparted tolerance against heat stress and improved the morphological features such as root shoot length, root number, fresh and dry weight, leaf area, and number [132]. Furthermore, application of NPs such as ZnO regulated the antioxidative system and chilling response transcription factors under chilling stress in *Oryza sativa* L. [133].

### 5.4. Metal/Metalloid Toxicity

Application of NPs are arising as a competent technique in the field of phytoremediation due to the effective interaction of the NPs with plants’ metabolism and metal ions. Phytoremediation is a sustainable technique for the removal of hazardous wastes from environment using potent plant candidates [134]. The NPs promoted growth of different plant species exposed to heavy metal toxicity by mitigating the oxidative stress elicited by heavy metals [111,135]. Application of 100 μM silicon dioxide improved the Cd, Cu, and Mn stress tolerance potential of *A. pygmaea* by augmenting biomass accumulation and increasing the activities of different biocatalysts in the plant [111]. Moreover, the silicon dioxide increased the absorption and accumulation of heavy metals in roots and thus prevented the translocation of the toxic compounds to the leaves [111]. NPs have the ability to immobilize the toxic metal ions and nanofibrous composite membranes using polyvinyl alcohol, and polyacrylonitrile have the metal chelation efficiency that aids in the removal of Cr and Cd [136]. This study also validated the metal chelation efficiency of NPs depends on the positive or negative charge it possesses on the surface [136]. The NPs have the potential to protect the membrane of the plant exposed to stress by preventing the membrane degradation through low MDA accumulation of NPs- treated plants exposed to metal stress [90]. In *Leucaena leucocephala*, ZnO NPs induced elevation of SOD, CAT, and APX activity that contributes to the reduction of MDA content under Cd and Pb stresses [90]. Addition of magnetic nano-Fe_3_O_4_ into the growing media of wheat seedlings contaminated with Pb, Zn, Cd, and Cu (10 mM) increased the activity of SOD and POD, and thus alleviated the MDA accumulation [135]. Fe NPs which upregulate the activity of antioxidant enzymes and glyoxalase through the accumulation of phytochelatins and glutathione simultaneously resulted in the boosting up of the tolerance to arsenic in rice [110]. Exposure to NPs recovered the mineral acquisition and thus maintained the biosynthesis of photosynthetic pigments in finger millet [137]. Parallel responses were observed in *G. hirsutum* when it was treated with ZnO NPs for tolerating Cd and Pb stresses [138]. The potential of ZnO NPs in the clearing of HM- contaminated media was established in a study performed in rice [109]. 

### 5.5. Flooding

The plants exposed to prolonged anaerobic condition as a result of flooding stress exhibit growth retardation and severe loss in crop productivity. Protein metabolism plays a significant role in the flooding stress tolerance of plants. Application of Ag NPs augmented the stress tolerance potential of soybean seedling by downregulation of protein mis-folding induced by flooding stress [112]. During flooding stress, augmentation of glyoxalase II 3, alcohol dehydrogenase 1, and pyruvate decarboxylase 2 genes was noticed, whereas upon the exposure of Ag NPs, the flood-induced metabolic changes were regulated and it reflected on the downregulation of all these enzymes [112]. Influence of Ag NPs in the production of the glyoxalase II 3 was one of the prominent outcomes of proteomics and this enzyme is considered as an indicator of cytotoxicity. When nicotinic acid and potassium nitrate (KNO_3_) were incorporated with Ag NPs, it further boosts up the flood tolerance in plants [114]. Another metal NP of Al_2_O_3_ also showed significant contribution in flood stress tolerance of soybean [115]. Moreover, NPs aid to fasten the recovery kinetics of flooding stress; soybean exposed to aluminum oxide nanoparticles (Al_2_O_3_ NPs) has the potential to recovery by the involvement of S-adenosyl-l-methionine-dependent methyltransferases and enolase [139]. The findings from the study conducted by Mustafa and Komatsu [115] give clear indication on the influence of size of NPs in flood tolerance, rather than the quantity and types. Three different sizes of Al_2_O_3_ NPs triggered different metabolic responses in plants under flood. The catalytic activity of isocitrate dehydrogenase was increased with the application of 5 nm Al_2_O_3_ NPs, but 30–60 nm Al_2_O_3_ NPs induced ribosomal protein production under flood. Whereas by the high concentration, 135 nm Al_2_O_3_ NPs, improved permeability of the mitochondrial membrane [115]. The differential imprints of 2, 15, and 50–80 nm Ag NPs on the tolerance mechanisms of the soybean under flood stress was reported by Mustafa et al. [140]. Of the three sizes, 15 nm Ag NPs was more effective due to the increase in ribosomal proteins, and amino acid metabolism-related proteins with a reduction in protein synthesis-related proteins. 

### 5.6. Other Abiotic Stresses

Apart from salinity, drought, temperature, and heavy metal stresses, other stresses such as high light, UV, and nutrient stresses can cause oxidative stress in plants, altering their growth and development. NPs such as TiO_2_ play a significant role in mitigating light stress by catalyzing the redox reaction, which leads to the generation of superoxide and hydroxide radicals. UV imparts negative consequences on growth as it induces oxidative stress. Photosynthetic apparatus would be highly damaged leading to ROS production and change in leaf structure following exposure to UV-B whereas application of SiNPs enhanced the antioxidant machinery to regulate oxidative stress resulting from UV-B exposure [8]. Thus, NPs modulate abiotic stress-induced responses at different levels in plants, and may be considered as potential tools for abiotic stress management in crops.

## 6. Dose-Dependent Variability of the Nanoparticle Action

Entry of NPs into the plant cells occurs via roots and leaves, and cause differential morphological and physiological changes, which either become inhibitory or stimulatory, depending upon the NPs’ properties, such as: chemical nature, size, reactivity, and the concentration of NPs. The inhibitory impacts of metallic NPs are apparent through its toxicity in plants. A number of research studies on plant–NPs interaction shows that NPs have both negative and positive effects, depending on the specific properties of NPs, their concentrations, reactivity, and plant species [141,142,143,144,145]. For instance, Lin and Xing [146] showed that seed supplemented with ZnO NPs at high concentration of 2000 mg L^−1^ negatively affected the germination of corn and ryegrass. Similarly, Ma et al. [147] observed the impacts of gadolinium (III) oxide (Gd_2_O_3_), cerium (IV) oxide (CeO_2_), ytterbium oxide (Yb_2_O_3_), and lanthanum (III) oxide (La_2_O_3_) at high concentration on the growth of cabbage, lettuce, radish, rape, cucumber, tomato, and wheat, and propounded that CeO_2_ inhibited the root elongation of lettuce at the concentration of 2000 mg L^−1^, while La_2_O_3_, Gd_2_O_3_, and Yb_2_O_3_ at 2000 mg L^−1^ suppressed the root elongation of all these seven plant species. Likewise, seed treated with TiO_2_ and aluminum oxide (Al_2_O_3_) affected seed germination, growth, and development of tobacco plants. A study of other researchers also showed the reduced growth of *C. annum* seedlings supplemented with 1 mg L^−1^ Ag NPs [148]. Inhibition of *Lemna minor* growth and the decreased activity of POX, CAT, and SOD activity were reported under CuO NPs (200 mg L^−1^) [149]. Moreover, ZnO NPs significantly declined the biomass of rye seedlings as well as affected the root anatomy by shrinking root tip, epidermal, and cortex cell deformation [146].

Several studies have shown that NPs at concentrations below certain limits stimulates seed germination [150,151], and plant growth and development [152,153]. For developing the better understanding of NPs’ influence on plant growth, further studies could be done based on the types and concentration of NPs. 

Experimental findings of Suriyaprabha et al. [154] show that SiO_2_ promoted seed sprouting of maize seedlings by increasing the nutrient uptake. A study related to TiO_2_ NPs’ impacts on soybean plant resulted in increased germination by enhancing the activity of nitrate reductase. Moreover, the NP-treated seed has the capability of increased water uptake, better water utilization, and increased nutrient uptake from the soil [155]. ZnO NPs at low concentration (10–20 μg mL^−1^) reportedly enhanced the seed germination as well as stimulated the plant growth of soybean [119], onion [23], peanut [156], wheat [157], and in cluster bean, *Cyamopsis tetragonoloba* [158]. Furthermore, Kumar et al. [159] also stated that Au NP at 10 and 80 μg mL^−1^ increased the plant growth and yield as well as enhanced the number and leaf area along with chlorophyll and sugar content in *A*. *thaliana*. Reportedly, the addition of Ag NPs at 20–60 ppm stimulated the plant length of mustard, beans, and corn, and also increased carbohydrate, chlorophyll, and protein content in *B. juncea* [160,161]. In Table 3, we tried to show the positive and negative impacts of various nanoparticles on plants.

## 7. Priming with Nanoparticles: An Emerging Stress Elicitor

Seed priming is the most effective method for mitigation of stress tolerance and enhancement of crop production in plants [171]. Priming approaches are established to augment germination and seedling growth by changing seed vigor or physiological status of the seed [172,173]. In the recent few years, nanopriming method of seed priming with synthetic NPs gained significance in crop advancement owing to their small size and distinctive physicochemical properties of nanomaterials [174]. NPs, besides improving plant growth, also safeguard from various kinds of stresses. Heavy metals (HMs) are bound to the NPs’ surface due to its great surface area and lesser size, therefore decreasing its accessibility [2]. NPs can simulate the antioxidant enzyme activity in nano-enzymes, which can scavenge from oxidative stress [175]. Photosynthesis is a key metabolic process in plants and a highly vulnerable approach, which alleviates oxidative and osmotic stress, and its usual working can be sustained. In photosynthesis apparatus, photosystem II, RubisCo, and ATP are the chief goals under stress conditions [176,177]. The SiO_2_ NPs enhanced chlorophyll, transpiration rate, WUE, and carbonic anhydrase activity in *Cucurbita pepo* under salinity conditions [122]. Likewise, TiO_2_ alters the photoreduction activity and prevents linolenic acid in the electron transport chain. It also reduces the oxygen evolution rate of chloroplast [123]. Numerous stress responses are exhibited by plants like changes in molecular machineries, stress response gene expression, and generation of antioxidative enzymes, which helps to exhibit significant function in scavenging the plants in severe environmental conditions [178]. Plants guard themselves from osmotic stress by generating different organic osmolytes like polyols and trehalose, and diverse amino acids like glycine and proline. NPs provide sustenance to plants in mitigating this defense mechanism [179]. In stress situations, ROS are generated by cell organelles, and this is the sign of abiotic stress conditions. Plants are furnished with enzymatic apparatus to cope with oxidative stress levied by the environment [2]. 

Priming induces enhancement in amylases, lipases, and proteases enzyme activities that degrade macromolecules for growth and development of embryos. It also mitigates stress at the germination stage and eventually results in greater rates of seedling appearance and efficacious seedling formation [180]. These biological impacts provide assistance to farmers in that they decrease the time, fertilization, and expenditure of re-seeding. Nanopriming increases α-amylase activity in rice plants and ensuing greater soluble sugar concentration for supportive seedling growth. However, more ROS generation was found in germinating seeds of nanopriming treatment in contrast to control rice plants, indicating that both ROS and aquaporins exhibit significant function in increasing the seed germination [181,182]. Diverse approaches for nanopriming mediating seed germination were suggested, comprising formation of nanopores for augmented water uptake, restarting antioxidant systems, formation of hydroxyl radicals for cell wall relaxing, and nanocatalysts for rapid starch hydrolysis [181].

## 8. Biochemical Mechanism of Metal/Metalloid-Based Nanoparticles to Mitigate Abiotic Stresses

NPs are essential implements which act as nanofertilizers, pesticides, herbicides, etc., for the proper growth and development of plants under various environmental stresses, though the exact mechanisms in particular are still undiscovered [15]. It is believed that there are some biochemical mechanisms such as detoxification pathway, especially based on the activities of enzymatic antioxidants behind the mitigation process of stress-induced damage using NPs. The reactivity of NPs is dependent upon some essential factors like shape, size, composition, surface properties, stability, chemical properties, purity and production process, and most importantly, dose applied [183,184,185,186]. Additionally, the susceptibility of NPs to different environments are mainly due to the transformation of their configuration phase and oxidation process [187]. The core conformation of NPs may vary plant species to species and are dependent upon the changes of environments leading to alter their chemical and physical properties that eventually exert different responses [188]. Khan et al. [9] reported that metal/metalloid NPs can combat the adverse effects of abiotic stresses in crops. Generally, NPs’ uptake take place via plasmodesmata, and the translocation of NPs occurs via apoplast and symplast [189]. They also demonstrated that application of NPs enhanced biomass levels, chlorophyll contents as well as photosynthetic processes, antioxidant machineries, synthesis of osmolytes, and carbohydrate contents in plant cells. Beside these, when NPs enter into the plant cells, it not only promotes N_2_ levels and protein contents but also regulate the gene expression during both biotic and abiotic stresses [189,190]. According to Sharifi et al. [175], NPs can simulate the antioxidant defense system as nano-enzymes which restrict the production of ROS under stress environments. NP supplementation increased the activities of some enzymatic antioxidants viz., SOD, CAT, APX, POX, etc., and also boost up the levels of glutathione levels, proline levels, and the phytochelatin synthesis in plants [190]. Mahato et al. [191] also reported that NPs restrict the generation of oxidative stress by upregulating the antioxidant defense system under different stressed conditions viz., salt stress, temperature stress, drought stress, UV stress, etc. Thus, in this viewpoint, the enhancement of mentioned parameters due to NP supplementation are responsible for the increase in tolerability in plants under environmental stresses. 

**Figure 3:**
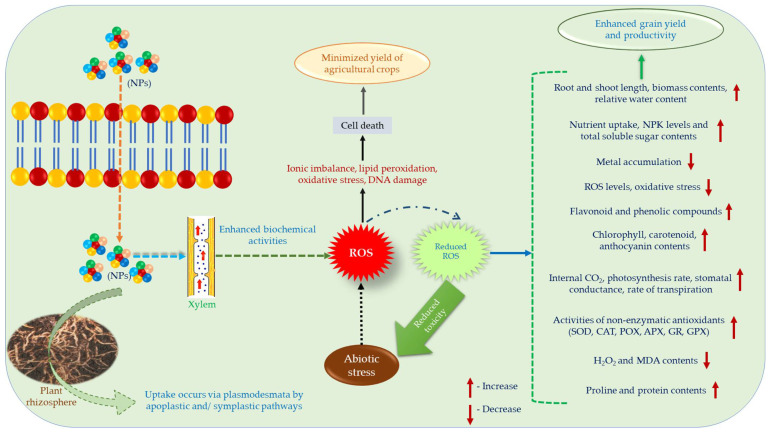
Schematic representation of uptake and impact of NPs during abiotic stress.

According to Liu and Lal [192] and Ranjan et al. [193], there are various kinds of NPs (viz., Mg NPs, TiO_2_ NPs, ZnO NPs, Cu NPs as CuO, Ag NPs as AgNO_3_, SiO_4_, Mn NPs as MnSO_4_, Ca NPs as CaCO_3_, Mo NPs, phosphorous NPs as [Ca_5_(PO_4_)_3_OH], AlO_4_ carbon nanotubes, Fe_2_O_3_ NPs, and chitosan complex of Cu or Zn) have been used in field conditions for proper growth and yield of agricultural crops. At first, NPs choose lateral root synapse to enter into the plant rhizosphere and outreach towards xylem via cortex and then pericycle [194]. However, their association with plants takes place on the basis of some biochemical activities which may activate not only the transport of ions into the cell but also reacts with -SH and -COOH groups, and modifies protein levels in the plant cells [195]. Additionally, NPs are able to form a network with the transporters present in the membrane of plant root cells to fetch inside the plants [196,197]. Thus, the transport of NPs into the cytoplasm occurs from roots to shoots, stem, leaves via cuticle, and ultimately in the grain but the main entrance is xylem [198,199]. Upon entry into the cell cytoplasm NPs form complexes with diverse cell organelles and consistently begin the metabolic pathways required for growth and yield of the plants [200]. In Figure 3, we have illustrated the effect of nanoparticles on abiotic stresses schematically, also, Table 4 lists the biochemical activities of some of the most common metal/metalloid-based NPs to combat the effects of abiotic stress.

## 9. Limitations of Using Nanoparticles for Crop Production

Though the supplementation with NPs caused positive impact on agricultural crops to mitigate various kinds of environmental stresses, all NPs cannot possess proper defense as it varies from species to species differentially [246]. There are several reports based on the NPs’ phytotoxicity that induced the synthesis of ROS and oxidative damage [198,247,248,249,250,251]. According to Gottschalk et al. [252] and Navarro et al. [253], the application of NPs in high dose caused toxicity whereas in low dose, NPs contributed a positive effect in combating abiotic stress-induced oxidative damage through antioxidant defense system [254,255]. NPs executed harmful effects by producing genotoxicity and oxidative stress in plants [146,247,256,257,258,259] that also affected the physicochemical metabolic pathways [94] by hampering the mineral uptake in agricultural crops [260]. The toxicity of NPs is dependent on not only the dose applied but also on the application process and its shape and size [251,261,262]. According to Manke et al. [263], the conformational alteration in shape and size of the NPs can lead to ROS production by affecting biochemical metabolism. They also demonstrated that the phytotoxicity of NPs is responsible for severe physiological deterioration by inducing inflammation, cell signaling, and genotoxicity. Ebbs et al. [251] reported that in plants, the toxicity levels of NPs regarding uptake, accumulation, and transportation also rely on the composition and surface area. Metal/metalloid-based NPs trigger Fenton reactions to generate free radicals that eventually produce ROS in plants [264]. There are some factors that are responsible for an imbalance of redox status of NPs, as a result, the antioxidant defense system would be downregulated and the generation of free radicals would be enhanced [265]. Priester et al. [266] stated that further investigation on the degree of NPs’ toxicity is vital for NPs’ supplementation in crops. Their uptake and accumulation should also be examined for better understanding. Therefore, keeping in mind these limitations, all the factors viz., size, shape, composition, surface area, application procedures, redox state, applied dose etc., should be investigated properly before application of NPs in agricultural fields to avoid ecotoxicological risks for both plants and humans.

## 10. Conclusions

Crop production globally has undergone several challenges in terms of climate and stresses. To overcome such challenges, nanotechnology has come up as a key component for sustainable development. Nanomaterials have the properties to nullify the harmful effects of abiotic stresses in plants by activating the antioxidant defense system of plants. Due to their property of being able to penetrate in plants and large surface area, they have more effective adsorption and targeted delivery, can be responsible in regulating photosynthetic efficiency and water uptake, and detoxifying reactive oxygen species, thereby enhancing seed germination, growth, and yield of crops. By careful analysis of dosage to be used for different nanomaterials, they can be sustainably utilized in the agriculture for better productivity. However, there is still a need for the risk assessment and fate of nanomaterials in plants and soil as well as their interaction with the ecosystem.

## Figures and Tables

**Table 1:** Categories and types of nanoparticles.

Categories of Nanoparticles	Types of Nanoparticles	References
Metal-based NPs	Gold, copper, aluminum, iron, silver, platinum, palladium	[21,22]
Metalloids NPs	Selenium, silicon, boron, arsenic, tellurium	[23,24]
Metal magnetic NPs	Cobalt, manganese, nickel, iron	[25,26]
Metal-oxide NPs	Titanium dioxide, cerium oxide, iron oxide, aluminium oxide, zinc oxide, copper oxide	[27,28]
Dendrimers	Hybrid, tecto, micellar, chiral, liquid crystalline, triazine	[29,30]
Carbon-based NPs	Carbon nanotubes, carbon nanohorn, nanodiamond, fullerene, graphite, graphene, graphene oxide, carbon dot	[31,32]

**Table 2:** Positive effect of various types of nanoparticles on some plant species under different abiotic stress conditions.

Plant Species	NPs	Concentration of NPs	Type of Stress	Response	References
*Mentha piperita* L.	Fe_2_O_3_	0, 10, 20, and 30 µm	Salinity	Decreased accumulation of proline and ROS	[99]
*Capsicum annum* L.	MnNPs	0.1, 0.5, and 1 mg L^−1^	Salinity	Redistributed manganese, sodium, potassium, and calcium content in shoot and root	[100]
*Solanum lycopersicum*	CuNPs	50, 100, and 150 mg L^−1^	Salinity	Increases lycopene, carotenoid, and SOD activity	[101]
*Triticum aestivum*	AgNPs	1 mg L^−1^	Salinity	Increased IBA, NAA, and BAP accumulation	[102]
*Lupinus termis*	ZnO	20–60 mg L^−^^1^	Salinity	Modulate growth, photosynthesis, and antioxidant responses	[19]
*Zea mays* L.	CuNP	3.33, 4.44 and 5.55 mg L^−^^1^	Drought	Higher biomass grain yield	[103]
Fragaria×ananassa Duch	Fe_3_O_4_	0.8 ppm	Drought	Improved morphological and growth parameters	[104]
*Glycine max*	CeO	0, 10, 100 and 500 mg kg^−1^	Salinity	Higher photosynthetic rate, RuBisCo carboxylase, and water use efficiency	[105]
*Gossypium hirsutum* L.	Graphene	200 µg ml^−1^	Drought	Increased fiber biomass	[106]
*Triticum aestivum* L.	TiO_2_	0.01–0.03%	Drought	Higher amount of gluten and starch	[107]
*Sorghum bicolor* L.	SeNP	10 mg L^−^^1^	Heat	Improved integrity in thylakoid and photosynthetic apparatus	[79]
*Lycopersicum esculentum*	SeNP	4–12 µM	Low and high temperature	Better morphological growth traits	[108]
*Oryza sativa*	ZnO NPs	5, 10, 15, 20 and 25 mg L^−^^1^	Cu and Pb	Reduced metal uptake	[109]
*Oryza sativa*	FeNPs	0.4–0.8 mg L^−1^	Arsenic stress	Reduced As uptake and oxidative stress	[110]
*Arundinaria pygmaea*	Silicon dioxide NPs	100 μM	Cu and Mn	Improved growth, photosynthesis and the action of protective enzymes	[111]
*Glycine max*	AgNPs	2 mg kg^−1^	Flood	Downregulated *alcohol dehydrogenase 1* and *pyruvate decarboxylase 2* genes	[112]
*Zea mays*	poly(epsilon-caprolactone)	2.5 kg ha^−^^1^	Herbicide toxicity	Reduced the mobility of atrazine in the soil and genotoxicity	[113]
*Glycine max*	Ag NPs	5 mg kg^−1^	Flood	Prevented mis-folding of proteins	[114]
*Glycine max*	Al_2_O_3_ NPs	50 mg kg^−1^	Flood	Regulated the AsA/GSH pathway and increased ribosomal proteins	[115]

**Table 3:** Dose-dependent impacts of nanoparticles on different plant species.

Type of Nanoparticle	NPs Concentration	Target Plant Species	Nanoparticles Impact on Plants	References
Positive impacts				
Copper NPs	69.4 µM	*Zea mays* L.	Increased leaf water content, biomass, anthocyanin, chlorophyll (Chl), and carotenoid contents. Controlled production of ROS and increased seed number, and yield.	[103]
Zinc-oxide NPs	50 and 100 ppm	*Solanum melongena* L.	Enhanced growth parameters, fruit yield, water productivity, and photosynthetic efficiency.	[162]
Titanium dioxide NPs	60 ppm	*Zea mays* L.	Increased growth regulating parameters, relative water content, potassium ion concentration, total phenolic content, proline content, and level of antioxidant enzymes.	[163]
Silicon NPs	300–1200 mg L^−1^	*Triticum aestivum* L.	Enhanced growth parameters and chlorophyll content. Optimized level oxidative enzymes. Increased plant biomass and yield.	[164]
Iron (III) oxide NPs	10, 50 and 100 mg L^−1^	*Sorghum bicolor* (L.) *Moench*	Improved and increased seed germination rate, seedling growth, photosystem II efficiency, Chl index, photosynthetic rate, and relative water content.	[165]
Negative impacts				
Silver NPs	80 and 160 mg L^−1^	*Pisum sativum* L.	Decreased seed germination and growth parameters. Deformation in root cells and caused increased chromosomal abnormalities.	[166]
Aluminum oxide NPs	50–1000 mg L^−1^	*Glycine max*	Damaged root surface and root cap.Altered lignin monomer composition and cell-wall esterified hydroxycinnamic acids. Reduced phenylalanine ammonia-lyase activity in stems.	[167]
Zinc oxide NPs	300, 600, and 1000mg kg^−1^	*Solanum lycopersicum* L.	Increased root uptake of zinc. Increased oxidative stress by overproducing H_2_O_2_ and reduced level of antioxidant enzymes (APX and SOD) also caused reduction in total phenols, flavonoids, β-carotene, and lycopene in fruits.	[168]
Ceria NPs	50, 100, and 200 mg kg^−1^	*Phaseolus vulgaris*	Increased stomatal conductance. Decreased antioxidative defense. Induced lipid peroxidation in root and fresh weight.	[169]
Silica NPs	250 and 1000 mg L^−1^	*Arabidopsis thaliana*	Reduced growth and development of seedlings. Caused chlorosis in leaves.	[170]

**Table 4:** Biochemical activities of some metal/metalloid-based NPs to combat abiotic stress effects.

Nanoparticles (NPs)	Abiotic Stresses	Impact on Plants to Mitigate Stress/to Enhance Tolerability	Plant Species	References
Si NPs (SiO_2_)	Mercury	Enhanced growth, chlorophyll levels, and decreased Hg accumulation in both roots and shoots	*Glycine max* L.	[201]
	Drought and salinity	Increased leaves’ growth and chlorophyll levels maintained an equilibrium between Na^+^ and K^+^ ions, promoted photosynthesis process	*Musa acuminata*	[202]
	Salinity	Increased growth, relative water content (RWC), proline contents, chlorophyll contents	*Fragaria* sp.	[127]
	Salinity	Regulation of salt toxicity-associated genes, elevated seed germination efficiency, root growth and weight	*Solanum lycopersicum* L.	[126]
	Drought	Increased biomass contents, photosynthetic pigment levels, and upregulated photosynthesis process by improving rate of net photosynthesis and conductance of stomata	*Crataegus* sp.	[117]
	Chromium [Cr(VI)]	Enhanced growth, nutrient uptake, and antioxidant enzymes’ activities reduced Cr(VI) accumulation	*Pisum sativum* L.	[50]
	Salinity	Increased RWC, crop yield, and the activities of enzymatic antioxidants	*Vicia faba* L.	[203,204]
	Cold	Inhibited seed dormancy, increased seed germination, and weight of seedlings	*Agropyron elongatum* L.	[205]
	Salinity	Enhanced growth parameters, proline levels, and pigment contents	*Ocimum basilicum*	[206]
	Salinity	Inhibited seed dormancy, increased seed germination, and fresh weight	*Lens culinaris* Medik.	[207]
	Salinity	Increased the rate of seed germination, growth; alleviated the levels of H_2_O_2_, MDA, electrolyte leakage; improved pigment contents and antioxidant defense system	*Cucurbita pepo* L.	[122]
	Salinity	Increased fresh weight, RWC, chlorophyll contents, and rate of photosynthesis	*Solanum lycopersicum* L.	[208]
	Salinity	Increased root growth, weight, seed germination	*Lycopersicum esculentum*	[209]
Ti NPs (TiO_2_)	Salinity	Enhanced germination, growth parameters of seedlings, fresh weight and dry weight, RWC, K^+^ ion, proline and total phenolic contents; also upregulated the activities of antioxidant enzymes and alleviated Na^+^ ion, MDA levels, and electrolyte leakage	*Zea mays* L.	[163]
	Drought	Elevated the dry weight of seedlings, RWC, chlorophyll, and carotenoid contents; also promoted transpiration rate and stomatal conductance	*Triticum aestivum*	[6]
	Arsenic (As)	Improved growth and biomass contents, reduced MDA contents, and induced the regulation of antioxidant properties	*Vigna radiata* L.	[210]
	Salinity	Positive impact on agronomically important attributes by inducing antioxidant properties	*Dracocephalum moldavica*	[211]
	Drought	Enhanced chlorophyll and carotenoid levels, reduced the accumulation of H_2_O_2_ and MDA	*Linum usitatissimum*	[212]
	Cadmium (Cd)	Inhibited the toxic effects of Cd, enhanced RWC, growth parameters, chlorophyll contents, rate of net photosynthesis; restricted lipid peroxidation and proline levels	*Glycine max* L.	[213]
	Cold	Upregulated the activities of RubisCo and phosphoenolpyruvate carboxylase, downregulated H_2_O_2_ content	*Cicer arietinum* L.	[214]
	Drought	Modulated toxic effects, improved biomass accumulation, and RWC	*Ocimum basilicum* L.	[215]
	Drought	Increased growth and starch contents	*Triticum aestivum* L.	[107]
	Cold	Reduced electrolyte leakage index and MDA contents	*Cicer arietinum* L.	[216]
Ag NPs	Salinity	Enhanced germination rate and no. of germinated seeds, downregulated the levels of oxidative stress, and induced the activities of antioxidant enzymes viz., APX, GR, GPX	*Triticum aestivum* L. cv. Pusa Kiran.	[217]
	Heat	Induced growth, area, and numbers of leaves	*Triticumaestivum* L.	[132]
	Salinity	Promoted growth and enhanced the synthesis of NAA, IBA contents, alleviated ABA level	*Triticumaestivum* L.	[102]
	Salinity	Increased seed germination rate, fresh weight, and dry weight	*Trigonella foenum-graecum*	[218]
	Salinity	Enhanced proline and carbohydrate levels	*Triticumaestivum* L.	[129]
	Cold	Upregulated the genes responsible for the activities of antioxidants	*Arabidopsis. thaliana*	[219]
	Flooding	Upregulated protein levels, growth parameters, and downregulated the production of toxic products in the process of glycolysis	*Glycine max*	[220]
	Dark	Enhanced pigments levels, activities of enzymatic antioxidants, reduced MDA level	*Pelargonium zonale*	[221,222]
	Post-harvest	Enhanced fresh weight and decreased bacterial colony formation in stem	*Chrysanthemum morifolium* L.	[223]
ZnO	Drought	Enhanced growth, RWC, and nutrient uptake	*Solanum melongena* L.	[162]
	Drought and cadmium (Cd)	Enhanced growth, chlorophyll contents, and SOD and POX activities	*Triticumaestivum* L.	[224]
	Salinity	Enhanced growth of both roots and shoots, biomass contents, chlorophyll contents, protein levels, photosynthetic parameters, and then, activities of CAT, SOD and POX	*Lycopersicon esculentum*	[225]
	Salinity	Upregulated protein and proline levels, enhanced the activities of antioxidants, reduced H_2_O_2_ and MDA levels	*Trigonella foenum-graecum*	[226]
	Arsenic (As)	Promoted growth and phytochelatin contents, decreased As uptake in the seedlings	*Oryza sativa* L.	[227]
	Salinity	Enhanced pigment contents, the activities of CAT and SOD; alleviated the levels of total soluble sugar and proline	*Abelmoschus esculentus* L.	[124]
	Arsenic (As)	Enhanced growth, reduced As uptake, increased photosynthetic activities, induced the activities of antioxidant enzymes	*Glycine max*	[228]
	Drought	Enhanced yield of grains and Zn accumulation	*Triticumaestivum* L.	[229]
	Salinity	Increased proline contents, total sugars, and the activities of CAT, SOD, and POX	*Mangifera indica* L.	[125]
	Drought	Enhanced antioxidant defense system and the synthesis of melatonin	*Zea mays* L. cv. Jidan 27	[57]
	Cadmium (Cd)	Enhanced growth, biomass contents, pigment contents, photosynthetic attributes, and the activities of antioxidant enzymes; alleviated Cd accumulation in shoots and roots	*Zea mays* L.	[230]
	Cadmium (Cd)	Enhanced growth, reduced Cd uptake and electrolyte leakage, induced the activities of POX and SOD	*Triticumaestivum* L.	[231]
	Cadmium (Cd) and lead (Pb)	Enhanced growth, pigment contents, protein levels, and antioxidant enzyme activities; reduced lipid peroxidation	*Lycopersicon leucocephala*	[232]
	Salinity	Enhanced growth, Zn levels, chlorophyll levels, rate of CO_2_ assimilation; reduced Na^+^ contents	*Helianthus annuus* L.	[233]
	Drought	Enhanced germination rate and reduced dry weight	*Glycine max*	[119]
Cu NPs	Drought	Enhanced biomass levels and productivity of grains, elevated chlorophyll, carotenoid and anthocyanin contents; reduced oxidative stress by upregulating antioxidant defense system	*Zea mays* L.	[103]
	Cadmium (Cd)	Enhanced growth and weight, decreased Cd accumulation, elevated ion contents and antioxidative properties	*Triticum aestivum* L.	[234]
	Chromium (Cr)	Enhanced growth and biomass contents, reduced Cr uptake, increased nutrient uptake and antioxidative properties	*Triticum aestivum* L.	[235]
Fe NPs	Drought and cadmium (Cd)	Enhanced growth parameters, photosynthetic activities, uptake of Fe; decreased Cd accumulation	*Triticum aestivum* L.	[236]
	Drought	Promoted H^+^-ATPase activity, maintained opening and closing of stomata; elevated biomass, pigment contents and internal CO_2_	*Arabidopsis thaliana*	[237]
	Chromium (Cr)	Restricted the conversion of Cr (VI) to Cr (III) and Cr (VI) accumulation	*Brassica juncea*	[238]
Fe_2_O_3_	Salinity	Decreased MDA and proline contents, subdued antioxidant properties	*Mentha piperita* L.	[99]
	Drought and cadmium (Cd)	Enhanced growth, biomass contents, nutrient uptake; upregulated antioxidant enzymes, photosynthetic attributes; reduced uptake and translocation of Cd	*Oryza sativa* L.	[239]
	Salinity and cadmium (Cd)	Promoted growth, plant weight, biomass and NPK contents; deceased Cd accumulation; elevated pigment contents and antioxidant enzyme activities	*Triticum aestivum* L.	[240]
	Drought	Enhanced growth and chlorophyll levels, decreased H_2_O_2_ and MDA levels	*Brassica napus*	[69]
Fe_3_O_3_	Salinity	Induced the production of flavonoid, phenolic compounds, and anthocyanin; enhanced the activities of APX, GR, CAT, and GPX	*Dracocephalum moldavica* L.	[241]
Fe_3_O_4_	Salinity	Promoted growth, pigment contents, RWC, total soluble sugar; enhanced membrane stability	*Fragaria x ananassa* Duch.	[104]
	Cadmium (Cd), lead (Pb), copper (Cu) and zinc (Zn)	Restricted the toxic effects of heavy metals, enhanced the activities of SOD and POX	*Triticum aestivum* L.	[135]
FeSO_4_	Salinity	Enhanced weight, pigment levels, photosynthetic attributes viz., net photosynthesis, stomatal conductance, assimilation of CO_2_, Fe concentration; decreased Na levels	*Helianthus annuus* L.	[242]
Al_2_O_3_	Flooding	Enhanced growth and induced biochemical activities	*Glycine max* L. *cv. Enrei*	[115]
	Flooding	Enhanced growth of hypocotyl, promoted protein levels in mitochondrial membrane, and glycolysis process	*Glycine max* L.	[112]
CeO	Salinity	Maintained ionic equilibrium, enhanced root growth, reduced the generation of ROS	*Gossypium hirsutum L.*	[80]
	Light, dark chilling and temperature	Enhanced internal CO_2_, quantum yield of PS-II, RuBisCo activity, and reduced ROS levels	*Arabidopsis thaliana*	[78]
CeO_2_	UV-B	Absorbed UV radiation and alleviated oxidative stress levels	*Chlorella vulgaris*	[243]
Chitosan NPs	Drought	Enhanced crop productivity, biomass contents, RWC, chlorophyll contents; promoted the rate of photosynthesis, and induced the activities of SOD and CAT	*Triticum aestivum* L.	[244]
	Drought	Enhanced RWC, weight and protein in grains, proline levels, and induced the activities of SOD and CAT	*Hordeum vulgare* L.	[245]

## Data Availability

All information is presented in this article.
